# Cytotoxic potential of *Anisochilus carnosus* (L.f.) wall and estimation of luteolin content by HPLC

**DOI:** 10.1186/1472-6882-14-421

**Published:** 2014-10-28

**Authors:** Jaykumar Bhagat, Richard Lobo, Nimmy Kumar, Jessy Elizabeth Mathew, Aravinda Pai

**Affiliations:** Department of Pharmacognosy, Manipal College of Pharmaceutical Sciences, Manipal University, Manipal, Karnataka 576 104 India; Department of Pharmaceutical Chemistry, Manipal College of Pharmaceutical Sciences, Manipal University, Manipal, Karnataka 576 104 India

**Keywords:** *A.carnosus*, Anticancer activity, BT-549, Luteolin, High performance liquid chromatography

## Abstract

**Background:**

*Anisochilus carnosus* (L.f.) wall (Lamiaceae), an annual herb which grows at high altitude is used extensively in folk medicine for the treatment of ailments such as gastric ulcer and skin diseases. The aim of our study was to evaluate the anticancer activity of different extracts of the leaves of *A.carnosus*. An attempt was also made to estimate the luteolin content in different extracts of *Anisochilus carnosus* by HPLC (High Performance Liquid Chromatography).

**Methods:**

In the current study, we explored the cytotoxic potential of petroleum ether, ethanolic and aqueous extracts of *A.carnosus* against breast adenocarcinoma cell line (BT-549), by *in vitro* MTT and SRB assay. We also detected the luteolin content in different extracts (ethanolic and aqueous) of *A.carnosus* by using HPLC as a tool of analysis.

**Results:**

The results demonstrate that petroleum ether and ethanolic extract of *A.carnosus* showed potent cytotoxic effect against BT-549 with an IC_50_ of 22.5 μg/ml (petroleum ether extract) and 87.24 μg/ml (ethanolic extract), by SRB assay, and 18.35 μg/ml (petroleum ether extract) and 58.64 μg/ml (ethanolic extract), by MTT assay. The aqueous extracts showed less cytotoxic effect with an IC_50_ of 211.26 μg/ml (by SRB assay) and 238.91 μg/ml (by MTT assay). HPLC results of luteolin content in various extracts using luteolin as the marker compound indicated the ethanol extract to contain the highest concentration of luteolin (0.372% w/w). The aqueous extract contained lower concentration of luteolin (0.282% w/w).

**Conclusion:**

Our findings demonstrate that petroleum ether and ethanolic extract of *A.carnosus* shows promising anticancer activity and has the potential to be developed into a therapeutic option for the treatment of cancer.

## Background

Breast cancer continues to be the second most common cause of cancer associated death among women [1]. It is projected that 1 in every 8 women will develop breast cancer in their lifetime [2]. Based on the ACS (American Cancer Society) report 2014, there are a possibility of 1,665,54 new cases of breast cancer and an estimated 585,720 people will die of this disease [3]. The risk factors for breast cancer include female sex, menarche at an early age, menopause at a late age, a family history of cancer, innate germ-line mutations in tumour-suppressor genes (e.g., BRCA1, BRCA2, and p53), late age at first pregnancy or never having given birth, regular alcohol use, use of menopausal hormone therapy (MHT) and ionising radiation exposure [1, 4]. Although, high dose chemo and radiotherapy have drastically increased the long term survival of breast cancer patients, they are associated with severe side effects such as nausea, vomiting, alopecia [5], cancer-related fatigue [6] and cardiovascular complications [1].

Several findings have indicated that more than two-third of human cancers could be averted through suitable lifestyle modification. According to Richard Doll and Richard Peto, 35% of death due to cancer is attributable to diet [7]. In this regard, nutritional phytochemicals and plant derived constituents such as flavonoids, phytosterols, triterpenoids and essential oils hold enormous possibilities for development of a therapy with considerably fewer side effects for treatment and prevention of cancer [8]. Studies have signified that they can alter multiple pathways at the same time and inhibit tumour promoting pathways while also activating tumour suppressor pathways [7, 8]. They can block or reverse two stages of tumour development namely tumour initiation and tumour promotion and thus terminate or impede the development of precancerous cells into malignant ones [7]. One such class of nutritional phytochemical that have been identified to have incredible cancer-protective effects is flavonoids. Luteolin is a naturally occurring flavonoid that can be found in a variety of fruits, vegetables and medicinal herbs [9]. Many epidemiological studies suggest a reverse relation between luteolin consumption and risk of some cancer types. *Anisochilus carnosus* (L.f.) wall, a member of the family Lamiaceae is an annual herb which grows on high altitude among small rocks. In India, it is widely distributed in Tamil Nadu, Karnataka, Maharashtra and Rajasthan [10] and is known as Induparni in Sanskrit, Panjiri-ka patta in Hindi and Kapurli in Marathi [11]. Traditionally, it is widely used by many tribal communities of Tamil Nadu, Maharashtra and Rajasthan for the treatment of cough, ulcer, eczema and stomach ache [12–16]. Phytochemical analysis of this plant showed it to be rich in active compounds such as flavonoids (luteolin, apigenin) [17], phytosterols, triterpenoids, saponins, tannins [18] and essential oil components (carvacrol, α-cis-bergamotene, caryophyllene, β-selinene, camphor) [19].

This paper reports the anticancer activity of different extracts of *A.carnosus* against BT-549, an estrogen independent breast adenocarcinoma cell line. This study also aims to determine the luteolin content in different extracts of the plant and correlate the luteolin content of extracts to its anticancer activity.

## Methods

### Plant collection

The leaves of *A.carnosus* was collected from Udyavar, Udupi in September 2010 and authenticated by Dr. Richard Lobo, Pharmacognosist, Manipal College of Pharmaceutical sciences, Manipal, Karnataka. A voucher specimen with accession number [PP 573] has been deposited in the Department of Pharmacognosy, Manipal College of Pharmaceutical Sciences, Manipal, India.

### Reagent and chemicals

The reagents MTT (3-(4, 5-dimethylthiazol-2-yl)-2, 5-diphenyltetrazolium bromide) and SRB (Sulforhodamine B) were purchased from Sigma Aldrich, USA. Methanol, ethanol (95%), chloroform, petroleum ether, acetone and benzene were purchased from Ranbaxy Fine Chemicals Ltd. The reference standard luteolin was obtained from Sigma Aldrich, USA. All solvents purchased were of analytical grade.

### Extraction and fractionation

The dried coarsely powdered leaves of *A.carnosus* (500 g) was extracted with solvents petroleum ether (60-80°C) (3x 1 L) and ethanol (3x1 L) using soxhlet extractor. The solvent- containing extracts were evaporated to dryness on a rotary evaporator under reduced pressure to obtain percentage yield of 23.8% w/w and 17% w/w respectively. Crushed shade-dried leaves (500 g) were kept for cold maceration in chloroform: water (1:99) for 4 days at room temperature. The solvent-containing extract was then filtered and filtrate obtained was concentrated on a water bath to obtain crude aqueous extract (16.2% w/w).

### Human cell line and culture medium

#### Cell line

Breast tumour carcinoma cell line BT-549 was obtained from NCCS (National Centre for Cell Sciences) Pune, India. DMEM (Dulbecco’s Modified Eagles Medium) media and Foetal bovine serum (FBS) was obtained from Sigma Aldrich, USA.

### *In vitro*MTT assay

The cytotoxicity of *A.carnosus* against BT-549 cell line was determined using the Methyl thiazol tetrazolium (MTT) assay [20]. BT-549 (Breast tumour carcinoma) cells were harvested from 75 cm^2^ tissue culture flasks and inoculated into 96-well flat bottom tissue culture plate (10^4^ cells/well in 0.1 ml of MEM [Minimum Essential medium] supplemented with 10% FBS [Foetal Bovine Serum]) and incubated for 24 hours for attachment. Test compounds were solubilised just prior to the experiment in 0.1% DMSO. After incubation for 24 hours, cells were subjected to several concentrations of the extracts (25, 50, 100 and 200 μg/ml). The cells which received only the medium containing 0.1% DMSO served as the control group. Paclitaxel was used as standard. After the treatment, the media was removed and washed with 200 μl of PBS. To each well of the 96 well plates, 100 μl of MTT reagent was added and incubated for 4 hours at 37°C. After 4 hours of incubation, the plate was inverted on a tissue paper to remove MTT reagent. To solubilize the formazan crystals in the wells, 100 μl of DMSO was added to each well. The suspension was placed on a micro-vibrator for 5 minutes and the optical density (O.D) was measured by a microplate reader at 570 nm. Percentage viability of each extract was calculated by using the formula:


The experiment was performed in three biological replicates. Results were expressed as Mean ± SEM values. % cell viability was plotted against the tested drug concentrations.

### *In vitro*SRB assay

Cell proliferation and viability was determined by a sulforhodamine B (SRB) colorimetric assay [21]. Briefly, BT-549 cells were plated onto 96-well flat bottom tissue culture plate at a density of 10^4^ cells/well and incubated for 24 hours. Subsequently, cells were treated with various concentrations of the extract (25, 50, 100, 200 μg /ml). The cells in the control group received only the medium containing 0.1% DMSO. Paclitaxel was used as standard. After 48 hours, the cells were fixed with ice-cold trichloroacetic acid (100 μl per well, 10% w/v) for 1 hour at 4°C. The plates were washed in distilled water and allowed to dry. 100 μl SRB (0.057% w/v in 1% aqueous acetic acid) solution was added to the dry 96-well plates in each well and allowed staining to occur at room temperature for 30 min. The sulforhodamine (SRB) solution was removed by washing the plate five times with 1% v/v acetic acid in order to remove unbound dye. Washed plates were then dried. 200 μl of 10 mM Tris Base (pH 10.5) was added to each well to solubilize the bound SRB. It was then shaken for 5-10 min on a shaker platform. Lastly, the plates were read in a 96-well plate reader with working wavelength of 570 nm. The O.D of SRB in each well is directly proportional to the cell number so the O.D values can be plotted against concentration and the IC_50_ determined. The viability and growth in the presence of test material is calculated by following formula:


### HPLC estimation of Luteolin

Samples for HPLC were prepared from the leaves of *A.carnosus* extract*.* 10 mg of ethanolic and aqueous extract were weighed and separately dissolved in HPLC grade methanol. They were filtered through a 0.2 μm syringe filter and transferred to 2 ml eppendorf tubes. The volume was made up to 2 ml with methanol to obtain 5 mg/ml concentration. These solutions were further used for HPLC estimation as test samples. Reference standard luteolin was prepared by weighing 0.1 mg/ml of luteolin and dissolving in 1 ml of HPLC grade methanol.

HPLC analysis was performed using Chen X *et al* method with slight modification [22]. SCL – 10A VP HPLC system (Shimadzu, Japan). Reverse phase separation was performed at room temperature 25°C using C-18 HYPERSIL column (250 × 4.6 mm), particle size 5 mm. The mobile phase consisted of 60% water i.e. (water containing 0.5% H_3_PO_4_ and methanol) (solvent A) and 40% Acetonitrile (solvent B). The flow rate was kept at 1 ml/min and the injection volume was 20 μl. The chromatogram peaks were detected at 254 nm. Detector used was SCL – 10A VP, UV-Visible (Shimadzu, Japan). The compounds were identified by comparing the retention times and peak areas of extracts with that of the reference standard luteolin.

### Statistical analysis

Graphs were prepared in Microsoft office 2010 beta – Excel. IC_50_ value was calculated using the linear regression equation in GraphPad Prism 5.0. Data was represented as mean ± standard error of mean of three samples. Statistical significance (*p*) was calculated by one-way ANOVA followed by Tukey’s post hoc test of significance where, *p* <0.05 was considered to be significant. Statistical comparison between the mean % viability of each extract and its negative control was done using one-way ANOVA followed by Dunnett’s post hoc test of significance wherein *p* <0.05 and *p* <0.01 were considered to be statistically significant and very statistically significant compared to negative control.

## Result

### *In vitro*MTT assay

**Figure 1:**
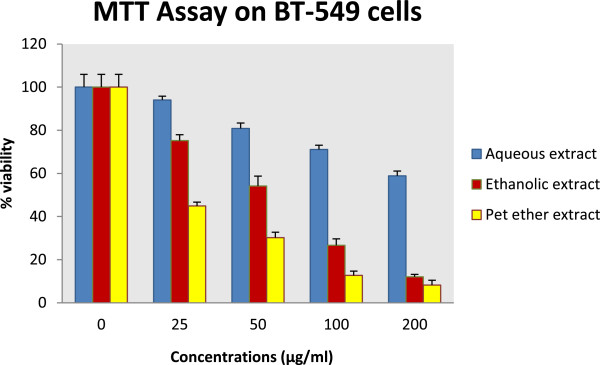
***In vitro*** **MTT assay on BT-549 cells.** BT-549 cells were incubated with different concentrations (25, 50, 100, 100 μg/ml) of aqueous, ethanolic and petroleum ether extracts of *Anisochilus carnosus*. Growth inhibition was determined by the MTT assay. The percentage of cell survival was calculated by defining the absorption of cells without *Anisochilus carnosus* treatment as 100%.

The ethanolic, aqueous and petroleum ether extracts of *A.carnosus* were evaluated for cytotoxicity against breast adenocarcinoma cell line BT-549 using MTT (3-(4, 5-dimethylthiazol-2-yl)-2, 5-diphenyltetrazolium bromide) assay. The cytotoxicity results clearly establish that the petroleum ether and ethanolic extract of *A.carnosus* exhibits potent cytotoxic effect against BT-549 with an IC_50_ of 18.35 μg/ml and 58.6 μg/ml. Further, both extracts showed dose-dependent inhibition of cell growth. The aqueous extract, conversely showed comparatively less pronounced cytotoxic effect with an IC_50_ of 238.9 μg/ml (Table 1 and Figure 1). Tukey’s post hoc tests indicated comparable cytotoxic activity between petroleum ether extract and paclitaxel as indicated in Table 1. Dunnett’s post hoc test displayed significant difference between the mean % viability of different concentrations of *A.carnosus* extracts and their corresponding negative control (DMSO).

**Table 1:** **Cytotoxicity results of** ***A.carnosus*** **extracts by MTT assay**

Concentration (μg/ml)	% Viability			
	Aqueous extract	Ethanolic extract	Pet. ether extract	Paclitaxel
0	100 ± 5.875	100 ± 5.875	100 ± 5.875	100 ± 2.09
6.25	-	-	-	80.32 ± 0.366**
12.5	-	-	-	42.56 ± 1.863**
25	94.04 ± 1.729	75.26 ± 2.654*	44.92 ± 2.631**	22.83 ± 1.22**
50	80.87 ± 2.462*	54.14 ± 4.610**	30.23 ± 3.662**	10.66 ± 0.46**
100	71.12 ± 1.950**	26.66 ± 2.951**	12.73 ± 4.242**	-
200	58.79 ± 2.278**	12.06 ± 1.104**	8.16 ± 1.063**	-
IC _50_ value	238.91	58.64^a^	18.35^a, b^	11.34^b^

Results were expressed as Mean ± SEM (n = 3) and analysed using one-way ANOVA followed by Tukey’s post hoc test of significance [where different alphabets denote significant difference (p <0.05)]. Statistical comparison between the mean % viability of each extract and its negative control was performed using one-way ANOVA followed by Dunnett’s post hoc test of significance wherein *p <0.05 and **p <0.01 were considered to be statistically significant and very statistically significant compared to negative control. *A.carnosus* extracts were tested at concentrations of 200, 100, 50, 25 μg/ml (they were not tested at concentrations of 6.25 and 12.5 μg/ml). Paclitaxel was tested at concentrations of 6.25, 12.5, 25 and 50 μg/ml.

### *In vitro*SRB assay

**Figure 2:**
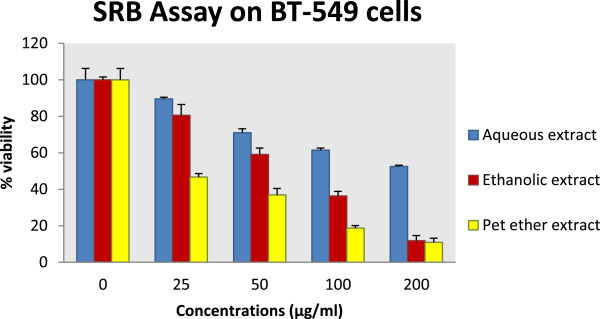
***In vitro*** **SRB assay on BT-549 cells.** BT-549 cells were incubated with different concentrations (25, 50, 100, 100 μg/ml) of aqueous, ethanolic and petroleum ether extracts of *Anisochilus carnosus*. Growth inhibition was determined by the SRB assay. The percentage of cell survival was calculated by defining the absorption of cells without *Anisochilus carnosus* treatment as 100%.

The ethanolic, aqueous and petroleum ether extracts of *A.carnosus* were evaluated for cytotoxicity against breast adenocarcinoma cell line BT-549 using SRB ( Sulforhodamine B) assay (Table 2 and Figure 2). Like MTT assay, results of SRB distinctly prove that petroleum ether extract and ethanolic extract of *Anisochilus carnosus* showed potent dose- dependent cytotoxic effect against BT-549 with an IC_50_ of 22.5 μg/ml and 87.24 μg/ml respectively. The aqueous extract, however showed less cytotoxic effect with an IC_50_ of 211.26 μg/ml. Tukey’s post hoc test revealed comparable cytotoxic activity between petroleum ether extract and paclitaxel. Dunnett’s post hoc test showed significant difference between the mean % viability of different concentrations of *A.carnosus* extracts and their corresponding negative control (DMSO).

**Table 2:** **Cytotoxicity results of** ***A.carnosus*** **extracts by SRB assay**

Concentration (μg/ml)	% Viability			
	Aqueous extract	Ethanolic extract	Pet. ether extract	Paclitaxel
0	100 ± 6.198	100 ± 6.198	100 ± 6.198	100 ± 2.44
6.25	-	-	-	86.25 ± 0.441**
12.5	-	-	-	45.36 ± 0.582**
25	89.50 ± 0.883	80.57 ± 1.972*	46.68 ± 5.838**	29.51 ± 1.54**
50	71.01 ± 2.141**	59.04 ± 3.507**	36.89 ± 3.525**	16.46 ± 2.313**
100	61.44 ± 1.129**	36.38 ± 1.396**	18.73 ± 2.505**	-
200	52.43 ± 0.745**	11.85 ± 2.371**	10.84 ± 2.741**	-
IC _50_ value	211.26	87.24	22.5^a^	13.91^a^

Results were expressed as Mean ± SEM (n = 3) and analysed using one-way ANOVA followed by Tukey’s post hoc test of significance [where different alphabets a, b denote significant difference (p <0.05)]. Statistical comparison between the mean % viability of each extract and its negative control was performed using one-way ANOVA followed by Dunnett’s post hoc test of significance wherein *p <0.05 and **p <0.01 were considered to be statistically significant and very statistically significant compared to negative control. *A.carnosus* extracts were not tested at concentrations of 200, 100, 50, 25 μg/ml (they were not tested at concentrations of 6.25 and 12.5 μg/ml). Paclitaxel was tested at concentrations of 6.25, 12.5, 25 and 50 μg/ml.

Overall, cytotoxicity of *A.carnosus* extracts against BT-549 cell line followed a similar trend by both MTT and SRB assay.

### HPLC estimation of Luteolin

**Figure 3:**
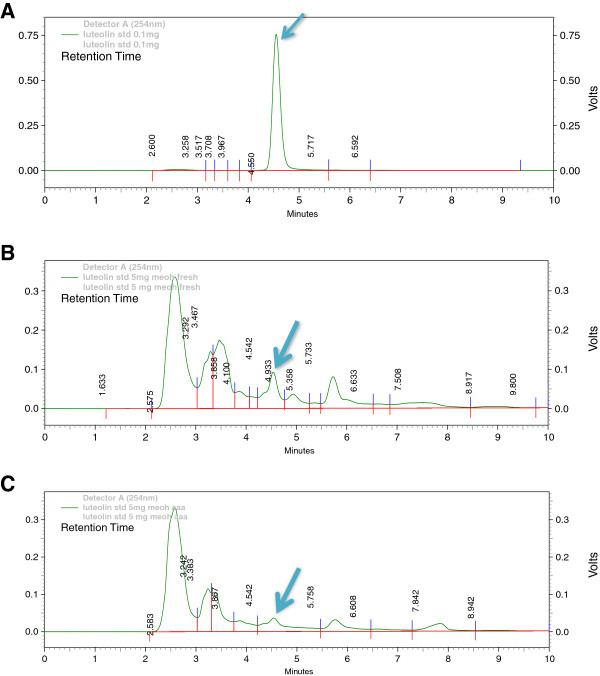
**Estimation of Luteolin in** ***A.carnosus*** **extracts.** Representative chromatograms illustrate High Performance Liquid Chromatography (HPLC) of luteolin **(A)**, ethanolic extract **(B)** and aqueous extract **(C)**. Absorbance was measured at 254 nm. Luteolin eluted at RT 4.5. The arrow marks indicate the location of luteolin peak.

The estimation of luteolin content in various extracts (ethanolic and aqueous) with luteolin as the marker compound was performed using HPLC (High performance Liquid Chromatography). Luteolin eluted at RT 4.542 in both ethanolic and aqueous extract (as can be seen in chromatograms in Figure 3A-C). The results shown in Table 3 and Figure 3(A-C) indicate that the ethanol extract of *A. carnosus* contains the highest concentration of luteolin (0.372% w/w). The aqueous extract contained lower concentration of luteolin (0.282% w/w).

The cytotoxic activity of ethanolic extract is probably due to the presence of luteolin. The presence of luteolin in ethanolic extract is evidently demonstrated through HPLC fingerprinting (Figure 3).

**Table 3:** **Estimation of Luteolin by HPLC**

S. no	Sample	Retention time	Peak area	Concentration (% w/w)
1	Luteolin	4.550	8385954	100
2	Ethanolic extract	4.542	1557755	0.372
3	Aqueous extract	4.542	1183180	0.282

## Discussion

Phytocompounds have been an excellent source of drug leads since several decades. They are unique in that they present an eclectic range of structural diversity and are by nature “biologically friendly” [23]. A recent survey of the European anticancer drug market revealed that of the 155 clinically approved anti-tumor drugs, 47% were either completely of natural origin or derived therefrom [24].

*A.carnosus*, a plant that grows commonly in high altitude regions has been attributed with innumerable benefits in folk medicine. Recently, a study has indicated nanoparticles synthesised from *A.carnosus* extract to show potent antimicrobial activity against microbes such as *B. subtilis*, *K. pneumonia*, *E.coli* and *P.aeruginosa* [25]. Azmathulla *et al.* [26] investigated the anti-ulcer property of *A.carnosus* and it was found to possess powerful gastric healing properties. Several reports have also shown *A.carnosus* ethanol extract to be an effective hepatoprotective agent [27, 28]. Furthermore, *A.carnosus* leaves showed good analgesic and antipyretic activity [29]. Such strong pharmacological activity of *A.carnosus* could be credited to the innumerable phytocompounds present in them.

In the present study, we have evaluated the cytotoxic potential of *A.carnosus* extracts using MTT and SRB assay. MTT assay establishes the cell viability on the basis of reduction of yellow tetrazolium MTT to purple formazan dye by mitochondrial dehydrogenase enzyme, an enzyme exclusively present in viable cells. As such, the quantity of formazan reveals the number of metabolically active viable cells [30]. The petroleum ether and ethanolic extract of *A.carnosus* showed potent cytotoxic potential against BT-549 with an IC_50_ of 18.35 μg/ml and 58.6 μg/ml respectively. Further, both extracts showed dose-dependent inhibition of tumor cell growth. The aqueous extract also displayed dose dependent cytotoxicity against BT-549 cell line with an IC_50_ of 238.9 μg/ml (Table 1 and Figure 1). According to the United States NCI plant screening program, a plant extract is usually considered to have active cytotoxic effect, if its IC50 value is 30 μg/ml or less [31]. Skehan and his co-workers conceived a novel method called Sulforhodamine B (SRB) assay to determine the cytotoxicity and cell proliferation based on the measurement of cellular protein content [32]. The petroleum ether and ethanolic extract of *A.carnosus* showed high cytotoxicity against BT-549 by SRB assay with an IC_50_ of 22.5 μg/ml and 87.24 μg/ml respectively. Additionally, both extracts exhibited dose-dependent inhibition of cell growth. The aqueous extract also demonstrated dose dependent cytotoxicity with an IC_50_ of 211.26 μg/ml (Table 2 and Figure 2). The mean % viability of all three extracts of *A.carnous* at concentrations of 50, 100 and 200 μg/ml was observed to be significantly different from their corresponding negative control (0.01% DMSO) by both cytotoxicity assays.

It was noted that IC_50_ values attained through MTT and SRB assay were highly comparable. *A.carnosus* aqueous extract gave a slightly higher IC_50_ value by MTT assay. IC_50_ values of ethanolic and petroleum ether extracts, on the other hand were found to be comparable though somewhat higher by SRB assay. Overall, there was good correlation between IC_50_ values obtained by both cytotoxicity assays, thereby confirming that *A.carnosus* extracts possess potent cytotoxicity.

We observed that aqueous extract of *A.carnosus* displayed comparatively less cytotoxic activity against BT-549 cell line than petroleum ether and ethanolic extract. Typically, a plant’s aqueous extract exerts cytotoxicity at comparatively higher concentration (approximately 250 μg/ml) than extracts obtained from other solvents such as methanol, ethyl acetate, ether etc. [33]. This is probably because water extracts in general, are thought to comprise of highly polar constituents such as carbohydrates, glycosides, proteins and minerals [34]. Moreover, phytochemical screening of *A.carnosus* successive water extract revealed the presence of constituents such as carbohydrates, glycosides, saponins and tannins in the water extract [18]. Specifically, phytosterols were absent in *A.carnosus* aqueous extract. As a general rule, phytosterols are insoluble in water, but soluble in non-polar solvents (like hexane, iso-octane and 2-propanol) [35]. Hence, the possibility of phytosterols being extracted into water is very less. Numerous studies have credited phytosterols with potent cytotoxic activity [36]. We presume the absence of phytosterols and terpenoids in aqueous extract could be a probable reason for low cytotoxic activity of water extract.

Reactive oxygen species (ROS), generated as a result of oxidative stress has been implicated in the development of cancer. ROS are capable of initiating signal-transduction pathways and inducing the transcription of proto-oncogenes such as c-fos, c-jun, c-myc, all of which are involved in accelerating the tumor growth [37]. Antioxidants, well-known for their high ROS scavenging potential are able to protect the cells from ROS attack and may thereby play a crucial role in the prevention of cancer [38]. The ROS scavenging potential of *A.carnosus* was investigated by Lobo R *et al* [39] and it was observed that the methanolic and aqueous extracts of *A.carnosus* exhibited potent antioxidant activity. We believe the high ROS scavenging potential of *A.carnosus* extracts could be a probable cause for its potent cytotoxic effect.

Several studies have indicated phytosterols to have tremendous antitumor potential. Being the counterparts of cholesterol in plants, they resemble cholesterol structurally, except for the C_24_ position on the sterol side chain [36, 40]. The anticancer effects of β-sitosterol, a dietary phytosterol have been documented in colon [41], breast [42] and prostate cancer [43]. In fact, 16 μmol/L β-sitosterol was able to induce cell death in MDA-MB-231 breast cancer cell line and LNCaP prostate cancer cell line four to six fold higher than control levels after 3-5 days of treatment [36]. In 1980, Raicht *et al.* [44] also reported that dietary β-sitosterol can provide protection from chemically induced colon cancer. They studied the development of methylnitrosourea induced tumor in rats fed with 0.2% β-sitosterol in their diet for 28 weeks. A remarkable decline was observed both in the number of rats that developed the tumor and the number of tumors per rat with β-sitosterol feeding. Besides, dietary supplementation of β-sitosterol at 60 mg/day for 6 months helped to improve the clinical symptoms of prostatic hyperplasia in humans [45], a disorder characterized by polyuria due to the enlargement of the prostate gland. For the same reason, epidemiologic studies propose a link between lower levels of prostatic cancer in Asians and vegetarians with diets rich in phytosterols compared with the Western diet [46]. Phytochemical screening of *A.carnosus* indicated the presence of phytosterols in the petroleum ether successive extract [18]. Consequently, the high cytotoxic activity of the petroleum ether extract reported in this study could be credited to the phytosterols present in *A.carnosus* petroleum ether extract*.*

Flavonoids are another group of nutritional phytochemical that has been widely acknowledged for its remarkable cancer-protective effects. Luteolin, a naturally occurring flavonoid exhibits powerful antitumor potential. Samy *et al.* [47] evaluated the anti-tumour potential of luteolin and cyclophosphamide combination against DMBA induced mammary carcinogenesis in Wistar rats and it was observed that the combination treatment reduced the incidence of tumour and decreased the tumour volume. Also, in a DMH induced colon cancer model, luteolin administration decreased the occurrence of colon cancer, total number and mass of tumors per rat by almost 90% in a dose-dependent manner both in initiation and post-initiation stages of colon carcinogenesis [48]. Luteolin isolated from *Terminalia arjuna* was found to inhibit proliferation of MCF-7breast cancer and HepG2 liver cancer cells in a dose dependent manner [49] *.* Luteolin also stalled proliferation of various tumour cell lines *in vitro* such as epidermoid, pancreatic, hepatoma, esophageal and lung cancer cell lines [50]. Hence, the potent cytotoxic activity of ethanolic extract of *A.carnosus* reported in this study can be attributed to its luteolin content. This is the first report on cytotoxicity of different extracts of *A.carnosus* against BT-549 breast adenocarcinoma cell line.

## Conclusion

The petroleum ether and ethanolic extracts showed remarkable cytotoxic activity against BT-549, an estrogen independent breast carcinoma cell line. It can thus be concluded that these extracts have the potential to be developed as a chemopreventive option for cancer. However, it is very difficult to conclude the possible mechanism for their cytotoxic activity at this stage. Hence, further research in this direction can be attempted to elucidate the molecular mechanism. Besides, they can be screened against other cancer cell lines for cytotoxic activity.

## Abbreviations

*A.carnosus*: *Anisochilus carnosus*
HPLC: High performance liquid chromatography
MTT: (3-(4, 5-dimethylthiazol-2-yl)-2, 5-diphenyltetrazolium bromide)
SRB: Sulforhodamine B
MEM: Minimum essential medium.
